# Laparoscopic Sigmoid Colectomy in a Patient With Congenital Left Renal Agenesis: A Case Report

**DOI:** 10.7759/cureus.109616

**Published:** 2026-05-25

**Authors:** Yuji Hidaka, Yasutomo Nagasue, Masao Uemura, Eiji Tanaka, Hiroshi Yokomizo

**Affiliations:** 1 Surgery, Japanese Red Cross Kumamoto Hospital, Kumamoto, JPN

**Keywords:** colorectal cancer, congenital renal agenesis, laparoscopic colorectal surgery, sigmoid colectomy, surgical case report

## Abstract

Congenital renal agenesis is a rare anomaly that can alter retroperitoneal anatomy and affect the spatial relationships between adjacent organs, potentially complicating colorectal surgery.

Here, we present the case of a 54-year-old woman with congenital left renal agenesis who was diagnosed with an obstructing sigmoid colon tumor. She initially underwent transanal decompression with an ileus tube and was referred to our institution for further management. Contrast-enhanced computed tomography revealed no distant metastasis and incidentally revealed the absence of the left kidney. After adequate decompression, a laparoscopic sigmoid colectomy with D3 lymph node dissection was performed. Preoperatively, we anticipated that identification of the retroperitoneal plane and mobilization of the splenic flexure would be technically challenging owing to the altered anatomy. However, intraoperatively, the retroperitoneal plane was identified without difficulty by maintaining continuity with the mesocolic plane and referring to consistent anatomical landmarks. By contrast, splenic flexure mobilization proved more technically demanding, as the colon was displaced into the left renal fossa, resulting in large distances from standard port positions, limited instrument reach, and suboptimal traction. Modification of port usage and the operator’s position enabled safe completion of the procedure without conversion to open surgery. The patient’s postoperative course was uneventful. Histopathological examination revealed a pT3N0M0 high-risk stage II adenocarcinoma, and adjuvant chemotherapy was administered. The patient remained alive without recurrence for 9.5 years after surgery.

In patients with congenital renal agenesis, retroperitoneal dissection may be simple despite altered anatomy, whereas splenic flexure mobilization can be more technically demanding owing to changes in spatial relationships. Careful preoperative assessment and flexible intraoperative strategies are essential for safe laparoscopic surgery in such cases.

## Introduction

Congenital renal agenesis is a relatively rare developmental anomaly with an estimated incidence of approximately one in 2,000 births. It can alter retroperitoneal anatomy and the spatial relationships between adjacent organs [1]. It is frequently associated with other congenital anomalies of the kidney and urinary tract as well as extra-renal malformations, reflecting its complex developmental background [1].

In colorectal surgery, surgeons rely on anatomical landmarks such as the ureter, gonadal vessels, and fascial structures to identify the retroperitoneal plane and mobilize the colonic flexures. However, these structures may be absent or altered in patients with renal agenesis.

Although laparoscopic right-sided colectomy in a patient with renal agenesis has been reported [2], the surgical implications of left renal agenesis in left-sided colon cancer remain unclear, particularly in terms of retroperitoneal dissection and splenic flexure mobilization.

Herein, we report a case of laparoscopic sigmoid colectomy in a patient with congenital left renal agenesis, focusing on intraoperative findings and technical considerations.

## Case presentation

In 2016, A 54-year-old woman presented with a one-day history of abdominal pain and was diagnosed with an obstructing sigmoid colon tumor at the referring hospital. Her medical history included hypertension and a prior cesarean section. She was initially managed with transanal decompression using a long tube. On day 4 after admission, she was transferred to our institution for further management.

Colonoscopy revealed a circumferential tumor causing luminal narrowing in the sigmoid colon (Figure 1).

**Figure 1:**
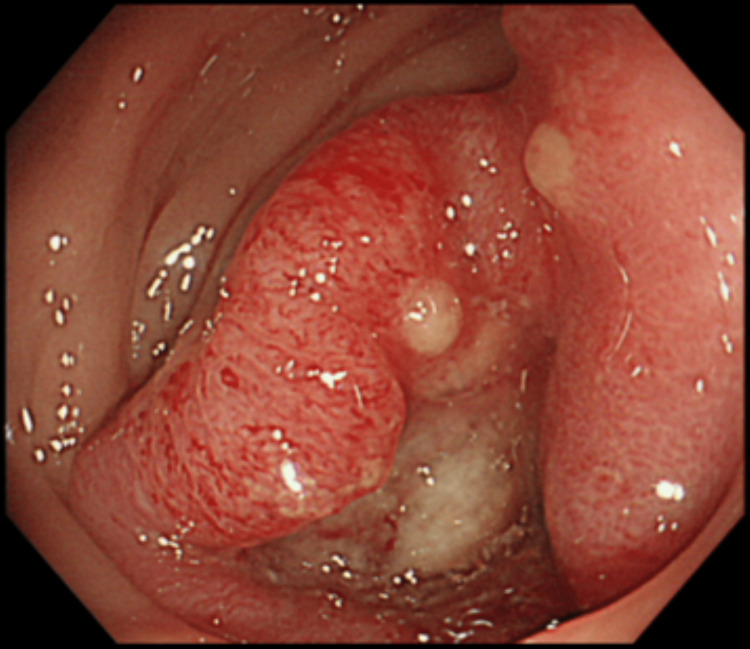
Colonoscopy. Circumferential tumor causing luminal narrowing in the sigmoid colon.

A contrast study demonstrated a stenotic lesion with proximal bowel dilatation (Figure 2).

**Figure 2:**
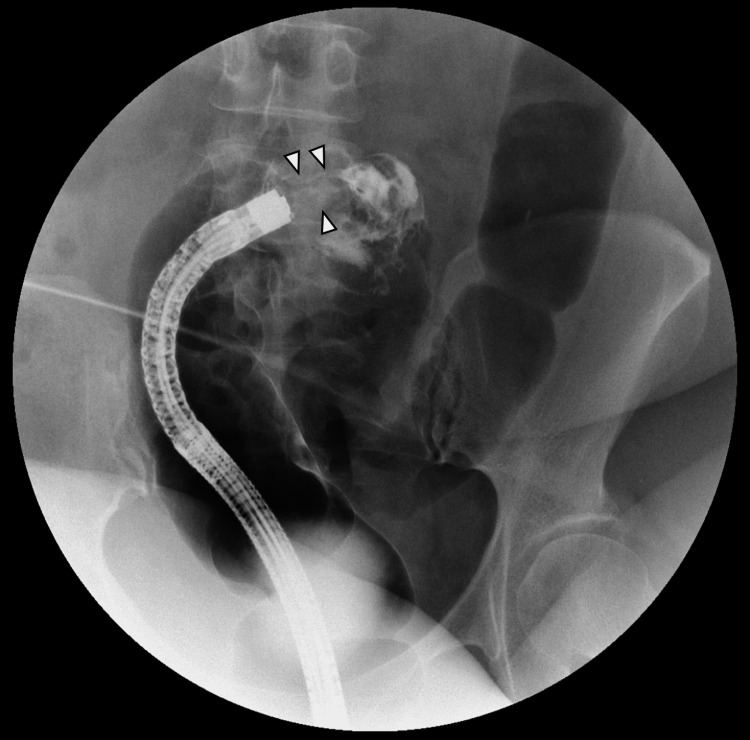
Contrast study. Stenotic lesion in the sigmoid colon with proximal bowel dilatation (arrowheads).

Contrast-enhanced computed tomography showed no evidence of distant metastasis and incidentally revealed the absence of the left kidney (Figure 3a), whereas the left adrenal gland was preserved (Figure 3b). The splenic flexure of the colon was displaced into the left renal fossa (Figure 3b). The patient’s kidney function was preserved despite this absence, with a serum creatinine level of 0.66 mg/dL (reference range, 0.47-0.79 mg/dL) and an estimated glomerular filtration rate of 71.9 mL/min/1.73 m^2^ (reference range, ≥60 mL/min/1.73 m^2^).

**Figure 3:**
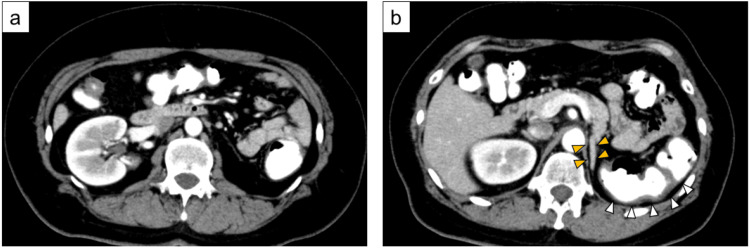
Contrast-enhanced computed tomography. (a) Absence of the left kidney. (b) The left adrenal gland is preserved despite the absence of the left kidney (yellow arrowheads). The splenic flexure of the colon is displaced into the left renal fossa normally occupied by the left kidney (white arrowheads).

After adequate decompression and preoperative evaluation, a laparoscopic sigmoid colectomy with D3 lymph node dissection was performed six days after the initial presentation. The operative time was 260 minutes, and blood loss was minimal.

The postoperative course was uneventful, and the patient was discharged on postoperative day 10. Histopathological examination revealed a well-to-moderately differentiated adenocarcinoma invading the subserosa without lymph node metastasis (pT3N0M0), corresponding to Stage IIA in the UICC TNM Classification (7th edition) [3] and Stage II in the Japanese Classification of Colorectal Carcinoma (8th edition) [4]. As venous invasion was present and has long been recognized as a high-risk feature in NCCN guidelines [5], adjuvant chemotherapy with uracil-tegafur plus leucovorin was administered for six months.

During follow-up, an incisional hernia developed and was repaired laparoscopically seven months after the initial surgery. The patient remained alive without evidence of recurrence 9.5 years after the colectomy.

Surgical technique

After induction of general anesthesia, the patient was placed in the lithotomy position. Five laparoscopic ports were placed in a standard configuration for sigmoid colectomy (an umbilical 12-mm camera port; bilateral lower quadrant 12-mm ports; bilateral lateral abdominal 5-mm ports; Figure 4).

**Figure 4:**
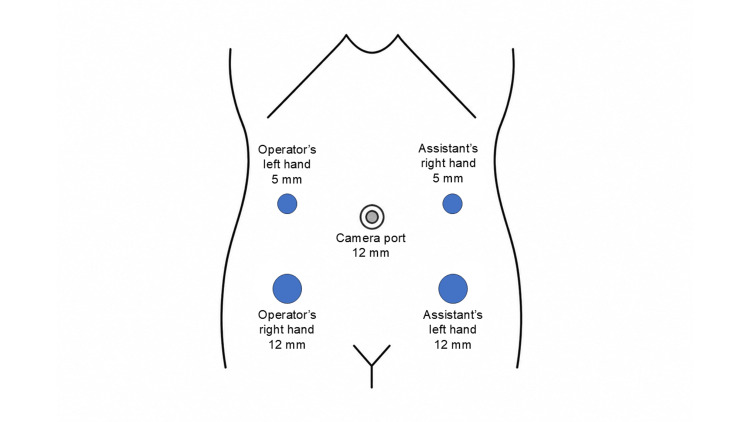
Schematic illustration of port placement for laparoscopic sigmoid colectomy. An umbilical 12-mm camera port, bilateral lower quadrant 12-mm ports, and bilateral lateral abdominal 5-mm ports are shown.

Based on preoperative imaging, we anticipated that identification of the retroperitoneal plane and mobilization of the splenic flexure might be technically challenging owing to the absence of the left kidney and altered anatomy.

Medial-to-lateral mobilization was initiated using a standard medial approach. Although typical landmarks such as the ureter and gonadal vessels were not identifiable, the boundary between the sigmoid mesocolon and the subperitoneal fascia was clearly delineated. This allowed reliable identification of the retroperitoneal plane, with the subperitoneal fascia serving as a key anatomical reference during dissection (Figure 5). Vascular ligation and D3 lymph node dissection were performed in the usual manner.

**Figure 5:**
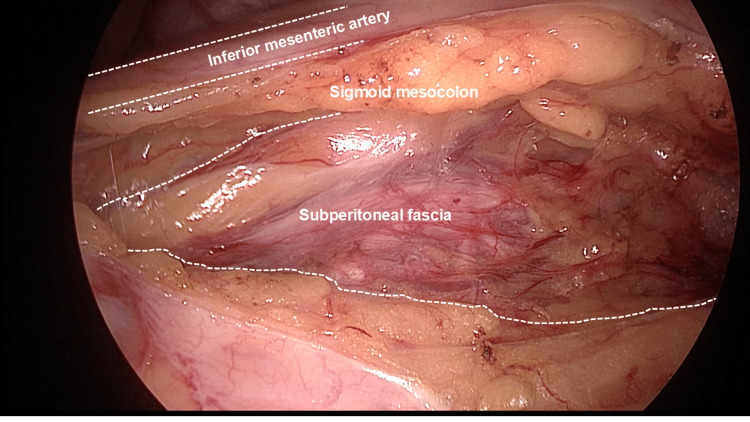
Intraoperative view during medial-to-lateral dissection. The boundary between the sigmoid mesocolon and the subperitoneal fascia is clear. The ureter and gonadal vessels were not identified during the medial-to-lateral approach.

In contrast to the relatively straightforward retroperitoneal dissection, splenic flexure mobilization was restricted by the altered anatomy. The splenic flexure was displaced into the left renal fossa, resulting in a large distance from the right-sided ports and limited instrument reach and traction angles (Figure 6).

**Figure 6:**
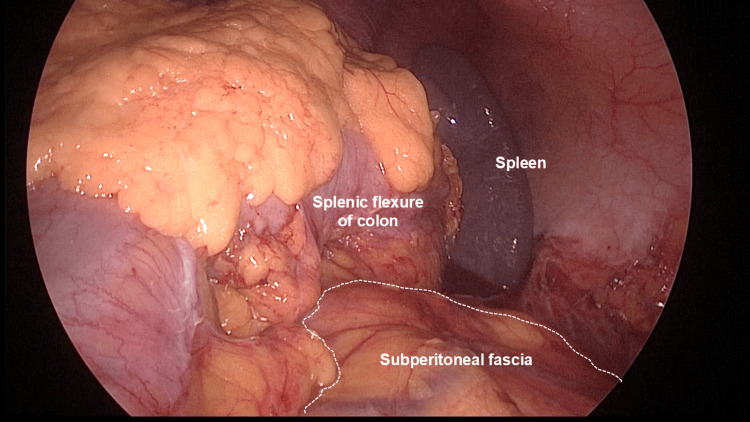
Intraoperative view during splenic flexure mobilization. The splenic flexure is displaced deeply into the left renal fossa, normally occupied by the left kidney. The subperitoneal fascia exposed during mobilization forms the retroperitoneal plane beneath the displaced colon. The descending colon was retracted toward the patient’s right side to facilitate exposure during mobilization.

To address this, the operator moved to a position between the patient’s legs and used the left-sided ports as the primary working ports. In addition, the assistant applied traction to the splenic flexure by using the right-sided ports (Figure 7). During the final stage of the lateral approach, a portion of the pancreatic tail was identified and preserved without injury. These adjustments improved visualization and countertraction, enabling safe mobilization of the splenic flexure.

**Figure 7:**
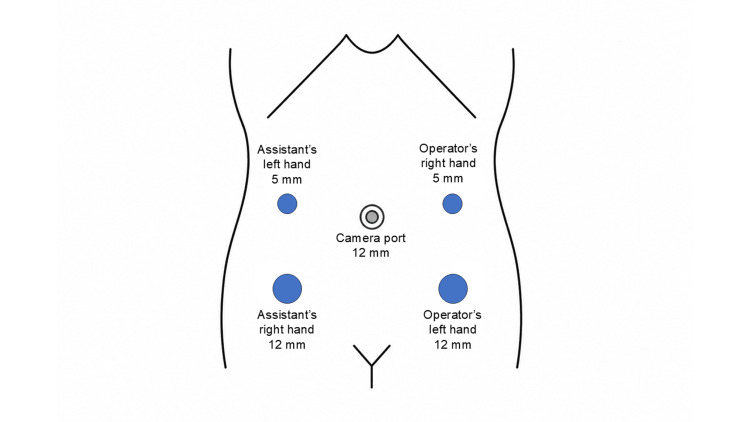
Schematic illustration of port placement for splenic flexure mobilization. The operator stands between the patient’s legs and uses ports on the patient’s left side, while the assistant uses ports on the right side to provide traction on the splenic flexure.

The specimen was extracted through a slight extension of the umbilical incision, and anastomosis was performed using a double-stapling technique without tension. Hemostasis was confirmed, and the procedure was completed without intraoperative complications.

## Discussion

In this case, we initially anticipated that identification of the retroperitoneal plane would be challenging because the patient’s ureter was absent and gonadal vessels might be difficult to identify owing to the altered anatomy. However, the retroperitoneal plane was identified without difficulty by maintaining continuity with the mesocolic plane and referring to anatomical structures other than those of the kidney, such as the subperitoneal fascia.

By contrast, splenic flexure mobilization proved the more technically demanding step in this case. Preoperative imaging suggested that the splenic flexure might be displaced into the left renal fossa owing to the absence of the kidney, which was confirmed intraoperatively. The altered anatomical relationship resulted in a large distance between the splenic flexure of the colon and the standard right-sided ports, limiting traction and instrument reach and thereby complicating laparoscopic mobilization.

Previous reports on laparoscopic colorectal surgery in patients with renal anomalies were largely focused on the associated anatomical variations, including ectopic and fused kidneys [2,6-8]. To the best of our knowledge, no previous report has described laparoscopic surgery for sigmoid colon cancer in a patient with congenital left renal agenesis. Such anomalies encompass a wide spectrum of positional, fusion, vascular, and developmental variations, including renal agenesis [1,9]. However, the influence of these anatomical differences on individual surgical steps has not been well described. This case suggests that congenital renal agenesis may have a differential impact on surgical steps: while retroperitoneal plane identification may remain simple, splenic flexure mobilization can be more technically demanding owing to altered spatial relationships.

Splenic flexure mobilization is an important step in left-sided colorectal surgery to achieve a tension-free anastomosis, as in this case, although its routine use remains controversial [10-12]. Although technical difficulty was anticipated owing to left renal agenesis, modification of the operator’s position and port placement allowed adequate visualization and successful completion without conversion to open surgery.

Although the tumor was classified as stage II, it exhibited venous invasion, a recognized risk factor for recurrence in stage II colorectal cancer [5]. The favorable long-term outcome in this case supports the appropriateness of standard oncological management in such patients.

This report is limited as it describes a single case, which limits the generalizability of the findings. Congenital renal agenesis is a relatively rare condition, complicating the accumulation of sufficient cases for broader validation. As anatomical variations may differ among patients, the findings of this case may not be universally applicable.

Overall, this case highlights that, in patients with congenital renal agenesis, retroperitoneal dissection can be performed safely by maintaining continuity with the mesocolic plane and using alternative anatomical landmarks, whereas splenic flexure mobilization may require technical modifications owing to altered anatomical relationships.

## Conclusions

Laparoscopic sigmoid colectomy can be safely performed in patients with congenital renal agenesis with appropriate recognition of altered retroperitoneal anatomy. In this case, retroperitoneal dissection was feasible by maintaining continuity with the mesocolic plane, whereas splenic flexure mobilization was more technically demanding owing to altered spatial relationships. Careful preoperative assessment and flexible intraoperative strategies are important to recognize anatomical variations and ensure safe surgery.

Further studies are warranted for a better understanding of the surgical implications of altered retroperitoneal anatomy in such patients.
